# The New Sydenham Society’s Publications for 1859

**Published:** 1860-07

**Authors:** 


### ART. IX.—*The New Sydenham Society's Publications for* 1859.

IT is well known to most of our readers that this Society, founded upon the ruins of the old Sydenham Society, established in 1843, went into active operation about two years ago, and that it has already issued several excellent works, edited for the most part in a very able manner, and executed in the highest artistic style. The publications for 1859 are the following:—

1. Diday on Syphilis in Infants and Children at the Breast. Translated by Dr. Whitley. 8vo., pp. 272.
2. Gooch on the Most Important Diseases of Women and Children. With other papers, wood-cuts, and a prefatory essay by Dr. Ferguson. 8vo., pp. 235.
3. Memoirs on Diphtheria, by Bretonneau, Guersant, Trousseau, Bouchat, Empès, and Daviot. Translated by Dr. Semple. With a Bibliographical Appendix by Mr. Chatto. 8vo., pp. 407.
4. Schrœder Van Der Kolk on the Spinal Cord, the Medulla Oblongata, and on the Proximate Cause and Rational Treatment of Epilepsy. Translated by Dr. W. D. Moore, of Dublin. With numerous lithographs. 8vo., pp. 291.

The names of the authors of these works are all familiar to fame.

The treatise of Gooch is one of the most readable and instructive monographs on the diseases of women and children in the English language, besides possessing the great merit of being comprised within remarkably narrow limits. The work of Diday, for many years principal surgeon to the Venereal Hospital of Lyons, is the most meritorious disquisition on infantile syphilis that has ever appeared in any age or country. The memoirs on diphtheria are by some of the most experienced and learned physicians of France, with Dr. Bretonneau, of Tours, by whom this malady was first described, at their head. Schrœder Van Der Kolk's volume is one of extraordinary interest and beauty, and alone is worth the subscription price of five dollars, the entire sum for the series. The plates are exquisitely executed.

We trust that the New Sydenham Society, which has so auspiciously commenced its career, will meet with adequate encouragement to enable it to proceed in its praiseworthy efforts of furnishing the profession with the reprints of some of the older English authors and the translations of French and German works now little accessible to the profession. American physicians should lend their aid in fostering and sustaining an enterprise which, in its effects upon the progress of science and humanity, may be rendered quite as useful in this country as among our British brethren.

